# Children with disorders of sex development: A qualitative study of early parental experience

**DOI:** 10.1186/1687-9856-2011-10

**Published:** 2011-10-12

**Authors:** Halley P Crissman, Lauren Warner, Melissa Gardner, Meagan Carr, Aileen Schast, Alexandra L Quittner, Barry Kogan, David E Sandberg

**Affiliations:** 1 Department of Pediatrics & Communicable Diseases Division of Child Behavioral Health University of Michigan Medical School 1500 East Medical Center Drive, SPC 5318 Ann Arbor, Michigan 48109-5318 USA; 2 Division of Urology The Children's Hospital of Philadelphia Richard D Wood Center, 3rd Floor 34th Street and Civic Center Boulevard Philadelphia, Pennsylvania 19104 USA; 3 Departments of Psychology & Pediatrics University of Miami 5665 Ponce de Leon Blvd. Coral Gables, Florida 33146-2070 USA; 4 Division of Urology Department of Surgery Albany Medical College 23 Hackett Boulevard Albany, New York 12208 USA

**Keywords:** disorders of sex development, qualitative, content analysis, psychosocial, health-related quality of life, genital surgery, parents

## Abstract

**Background:**

Clinical research on psychological aspects of disorders of sex development (DSD) has focused on psychosexual differentiation with relatively little attention directed toward parents' experiences of early clinical management and their influence on patient and family psychosocial adaptation.

**Objectives:**

To characterize parental experiences in the early clinical care of children born with DSD.

**Study Design:**

Content analysis of interviews with parents (n = 41) of 28 children, newborn to 6 years, with DSD.

**Results:**

Four major domains emerged as salient to parents: (1) the gender assignment process, (2) decisions regarding genital surgery, (3) disclosing information about their child's DSD, and (4) interacting with healthcare providers. Findings suggested discordance between scientific and parental understandings of the determinants of "sex" and "gender." Parents' expectations regarding the benefits of genital surgery appear largely met; however, parents still had concerns about their child's future physical, social and sexual development. Two areas experienced by many parents as particularly stressful were: (1) uncertainties regarding diagnosis and optimal management, and (2) conflicts between maintaining privacy versus disclosing the condition to access social support.

**Conclusions:**

Parents' experiences and gaps in understanding can be used to inform the clinical care of patients with DSD and their families. Improving communication between parents and providers (and between parents and their support providers) throughout the early clinical management process may be important in decreasing stress and improving outcomes for families of children with DSD.

## Introduction

In 2005, the Lawson Wilkins Pediatric Endocrine Society (renamed the *Pediatric Endocrine Society *in 2010) and the European Society for Paediatric Endocrinology convened a consensus conference on the management of "intersex" [1]. Conference participants recommended a new diagnostic nomenclature and introduced "disorders of sex development" (DSD) as the superordinate term for "congenital conditions in which chromosomal, gonadal, or anatomic sex development is atypical" [1].

Research on the psychological development of persons with DSD has focused on understanding the influence of atypical sex hormone exposure during steroid-sensitive periods of prenatal brain development on the process of psychosexual differentiation (i.e., gender identity, gender role, and sexual orientation) [2-5]. Analysis of clinical management strategies has focused on gender assignment and the desirability and timing of genital surgery [1,6-8].

The DSD Consensus Statement [1] recognizes that these conditions can exert substantial strain on the family; however, there have been relatively few systematic studies of how early interventions and interactions between healthcare providers and the family affect the quality of life of affected persons or their parents [9,10]. One study of parents of young children (predominantly 46, XY) with DSD found that 19% and 13%, respectively, reported clinically significant parenting stress and diminished adaptive coping capacity [11]. Interestingly, these self-report ratings were unrelated to the degree of masculinization of the child's external genitalia. Other evidence of the burden of the medical condition on the family was reflected in the observation that over 60% of these parents experienced difficulties in discussing their child's condition with relatives and friends and 68% were concerned that the DSD would result in their child being stigmatized [11].

Similar gaps in our understanding extend to parental reactions to early medical interventions [9,10]. Although early surgery may, in some cases, be necessary to allow for unobstructed urinary output without infections or to eliminate potential malignancy risk associated with dysgenetic gonads, early timing of procedures has also been justified as a strategy to relieve parental distress and reduce the likelihood of stigmatization, despite a lack of systematic evidence to support this belief [1,8,9,12,13].

Parental understanding of DSD pathophysiology and treatment options soon after the child's birth has received scant attention [13-15]. For example, parents' conceptualizations of the relationship between biological indices of sex development (i.e., karyotype, gonadal determination, sex hormone production and genital phenotype) and psychosexual differentiation remain largely unexplored. The same holds true for parents' views of the linkages between gender assignment and necessity of genital surgery, the benefits and drawbacks of disclosure of the child's DSD to extended family and friends, and parents' experiences with healthcare providers during the earliest stages of DSD ascertainment and clinical management.

Theoretical models of adjustment to congenital chronic medical conditions recognize critical parental influences on the affected person's adjustment during childhood and beyond [16-18]. Recent reports underscore the strain experienced by parents of newborns and young children born with DSD [14,19]; however, more complete information is needed regarding parents' experiences during the diagnostic and early decision-making periods associated with DSD. The goal of the present study was to identify clinically salient aspects of the parental experience regarding the diagnosis and clinical management of their children.

## Methods and Participants

### Design

A secondary data analysis was performed on interview transcripts from a study designed to develop health-related quality of life (HRQoL) measures for young patients with DSD and their families. The primary study was designed to evaluate the relevance, importance and clarity of preliminary quality of life items generated via open-ended interviews with parents, health care providers and advocates [20]. Items forming the provisional parent self-report HRQoL questionnaire were clustered into 10 subscales designed to capture both common and rare DSD-specific issues (*healthcare*, *decision-making*, *talking to others*, *role functioning/family activities*, *gender concerns*, *social functioning*, *general emotional functioning*, *medications*, *surgery*, *doctor visits*, *future concerns*, and *earliest experiences*). Parents completed the questionnaires in written form and then participated in cognitive interviews during which they were asked about their responses to each item using standardized prompts, including: "What did you think of when answering this question?" and "How did you decide on your rating?" [21,22]. Parents were asked to comment on any aspects of their experience that were not covered by the existing questions at the end of each subscale and at the end of the questionnaire itself. Structured interviews were conducted by clinician-researchers and experienced research staff (MG, ALQ, AS, DES, MB, and LC), trained by ALQ to conduct cognitive interviews. This research team has extensive experience in conducting these types of interviews [23,24]. Interviewers followed a structured protocol of open-ended questions and were instructed to allow parents to explore their thoughts and experiences without interruption.

Participant responses to cognitive interview questions often involved detailed descriptions of personal experiences related to the diagnosis and management of their child's DSD that went beyond responding to the standardized prompts. Interviews were audio-recorded and transcribed verbatim. Parents' responses to the items and follow-up interviews, together with demographic information and medical chart excerpts collected in the primary study, constituted the dataset for this secondary analysis.

All study procedures were approved by participating medical centers' Institutional Review Boards.

### Participants

Participants were parents of children with DSD identified by systematic medical record review at four regional medical centers located in metropolitan areas along the East Coast and Midwestern United States. Stratified, random sampling was employed to create a sample representative of a wide range of DSD diagnoses, phenotypic severity, gender assignment decisions, and age (newborn to age 6 years). Because of the sampling strategy adopted, the breakdown by diagnosis does not reflect the natural incidence of DSD. Genital surgery status was not used as a selection criterion. Participants who could not communicate in English or whose child had a documented, significant developmental delay (e.g., autism) were excluded.

Of the 134 households with an index child meeting study eligibility criteria, three were subsequently excluded based on details provided by parents: one child exhibited marked developmental delay and two parents were unable to be interviewed in English. One non-parent caregiver who contributed data to the primary study was also excluded. Of the remaining 130 index cases, recruitment continued until it was determined that successive cognitive interviews did not yield new information or new themes (i.e., saturation of content). A total of 19 of 130 eligible households (15%) refused to participate. The final sample comprised 41 parents (27 mothers and 14 fathers; Table 1) of 28 DSD-affected children (Table 2). Participants received $50 as compensation for their time.

**Table 1:** Parent participant characteristics (n = 41)

**Parenting Role**	**n (%)**
	
Mother	27 (65.9)
Father	14 (34.1)
**Racial Identification**	**n (%)**
	
Non-Hispanic	28 (68.3)
Hispanic	4 (9.8)
Black	3 (7.3)
Other	2 (4.9)
Declined to Respond	4 (9.8)
**Education**	**n (%)**
	
Partial High School	2 (4.9)
High School Graduate	8 (19.5)
Some College Education	11 (26.8)
College or University Graduate	9 (22.0)
Graduate or Professional Degree	7 (17.1)
Declined to Respond	4 (9.8)
**Yearly Family Income**	**n (%)**
	
< 20,000	8 (19.5)
20,000-40,000	9 (22.0)
40,000-60,000	5 (12.2)
60,000-80,000	4 (9.8)
> 80,000	11 (26.8)
Declined to Respond	4 (9.8)
**Age, *years***	
Mean (SD)	34.7 (6.7)
Range	19.5-50.8
Declined to Respond, n (%)	6 (14.6)

**Table 2:** Participants' children's characteristics (n = 28)

	**n (%)**			
**Gender**				
Boy	19 (67.9)			
Girl	9 (32.1)			
**Age (years)**				
Mean (SD)	4.1 (1.9)			
Range	0.75-6.99			
**Number of Genital Surgeries**				
Mean (SD)	2.46 (1.06)			
Range	1-4			
			**Gender of Rearing**	
			**Boy**	**Girl**
**DSD Diagnosis**^a^		**n (%)**	**n (%)**	**n (%)**
**Sex Chromosome DSD**		3 (10.7)	3 (100)	0 (0)
*45XO/46XY Mixed gonadal dysgenesis*		1	1	0
*45XO/46XisoY Mixed gonadal dysgenesis*		1	1	0
*45XO/46XY/47XYY Mixed gonadal dysgenesis*		1	1	0
**46, XX DSD**		8 (28.6)	1 (12.5)	7 (87.5)
*Congenital adrenal hyperplasia*		7	0	7
*Ovotesticular DSD*		1	1	0
**46, XY DSD**		17 (60.7)	15 (88.2)	2 (11.8)
*Aphallia*		1	1	0
*Complete androgen insensitivity syndrome (AIS)*		1	0	1
*Partial AIS (PAIS)*		1	1	0
*Partial gonadal dysgenesis*		3	2	1
*Partial AIS vs Idiopathic vs 5α-reductase-2 deficiency*		4	4	0
*Severe hypospadias with cryptorchidism and chordee*		1	1	0
*Severe hypospadias with chordee*		6	6	0

^a ^see reference [1] for details of DSD nomenclature.

Details regarding the child's medical history, including diagnosis and management, were excerpted from the medical record using standardized forms completed by qualified healthcare staff at participating medical centers.

### Data Analysis

All participants shared the experience of parenting a newborn and/or young child with DSD and associated medical management; thus, a phenomenological approach was well-suited to guide the qualitative content analysis [25]. Three investigators (HPC, LW, and MC) who were not involved with the design or evaluation of the primary study's HRQoL questionnaires, independently read the cognitive interview transcripts and highlighted parent dialogue that reflected salient thoughts, beliefs, and experiences related to their child's DSD and management. This process resulted in an initial outline of emergent categories. The same investigators then independently read the transcripts a second time to supplement and restructure the outline to better reflect parents' experiences. These outlines were merged through a process of comparison and data reduction. In contrast to the HRQoL questionnaire development process, in which we sought to capture the full range of experiences including those that were rare, this analysis focused on overarching themes expressed across multiple domains of the interview transcripts.

"Member checking" was used to improve the accuracy, credibility, and validity of the investigators' coding and interpretation of the cognitive interviews [26]: a convenience sample of study participants from one site was provided a draft of the study results for comment. Participants (n = 5) were asked to comment on the accuracy and completeness with which their experiences were represented. These respondents' comments were used to refine the results.

## Results

Four overarching and clinically salient domains of parental experiences rearing young DSD-affected children emerged from the content analysis: (1) sex announcement and gender assignment, (2) surgical decision-making, (3) sharing information about the child's DSD with others, and (4) interactions with healthcare providers. Parents who participated in the member-checking interviews identified these domains as accurately reflecting the major issues they experienced.

### Sex Announcement and Gender Assignment

A common feature of many DSD is atypical or ambiguous external genitalia. External genitalia ambiguity can be associated with a delayed gender announcement at birth (i.e. "It's a boy!" or "It's a girl!"). Although there is a well-recognized distinction between biologic "sex" (male/female) and "gender of rearing" (boy/girl), parents and providers often conflated the two constructs, e.g., "genetically, he was a boy." [27]

*Parental perspective*. Most parents voiced certainty about knowing their child's sex and gender of rearing immediately or soon after their child's birth-despite known discordance across karyotype, gonads, or genital anatomy. Parents used (1) their personal intuitions or "gut feelings," (2) the visible appearance of the child's external genitalia or imaging reports of the internal sex organs, and/or (3) genetic testing results to justify their conviction.

A number of parents expressed confidence about knowing their child's sex and gender prior to medical testing. One mother explained that she did not need testing performed on her child to know her child was a girl: "We never even doubted it." Another parent described feeling that they "didn't really have to assign" a gender, though doctors performed a laparoscopy and, in the parent's words, determined that the child "had more boy parts than girl parts" and thus, was a boy. Others cited karyotype findings as definitively determining both their child's sex and gender, erroneously assuming that sex chromosomes are the ultimate arbiters: "We had genetic testing done. So it wasn't like we had to choose and we had to worry about whether we were doing the right thing or not."

*Medical chart excerpts*. In contrast to parents' nearly immediate certainty about whether their children were males or females/boys or girls, a review of children's medical records revealed healthcare provider delays in gender assignment (n = 7), gender reassignment after initial birth announcement or assignment (n = 3), and cases in which karyotype did not match assigned sex or gender of rearing [n = 6, including 3 cases of sex chromosome mosaicism (e.g., 45, XO/46, XY; Table 2)].

### Surgical Decision-Making

*The necessity of surgery*. All children (n = 28) in this study had at least one genital surgery prior to parental participation in this study, though prior surgery was not an inclusion criterion. Reflecting on early decision-making, parents recalled strong wishes to surgically "normalize" their child's sexual anatomy, i.e., external genitalia and internal reproductive structures. Many parents viewed surgery as obvious and necessary, and did not experience it as something that involved a decision-making process. One parent stated: "The minute he was born, here he had this-- it has to be fixed... It was never any question whether he was gonna go through the surgery or not." Parents frequently used the term "fix" to describe how they understood various surgical interventions.

Parents expressed a profound trust in the medical team's recommendations: "We really never had to make a decision... the doctors told us what was gonna need to be done." As one parent explained: "I wanted them to do the best that they can for my son. So umm, anything they asked for or wanted to do, I was ok with it."

*Anticipated benefits*. Parents expressed a strong belief that surgery would (1) "fix" the appearance and function of their child's external genitalia and reproductive structures, and (2) avert expected negative psychosocial consequences associated with DSD (i.e. non-normative gender identity and/or gender role, teasing from peers, and hardship in future romantic relationships): "We felt [surgery] would be beneficial for health and social reasons like teasing in school." This sentiment was echoed by others: "In our son's case, he would have had to pee sitting down for the rest of his life and that has both social and physical aspects." Parents sought to surgically modify aspects of the DSD that they thought would be barriers to positive daily functioning: "We want him to have as normal a life as possible. So the benefits outweigh the risks." Parents perceived the medical team as reinforcing the idea that surgery would resolve the DSD: "Dr. [Urologist] was called in and, like a ray of sunshine, said 'I can fix this.'"

*Post-surgery experiences*. Some parents felt all early concerns related to their child's DSD were eliminated by surgery: "We don't even talk about it anymore. It's just not an issue for us anymore, you know. It's been repaired, and that's it." In general, parents did not report thinking about their children's DSD on a day-to-day basis: "It comes and goes in waves with us definitely... he has his surgery and everything and it's 'oh my gosh, it's kinda real again.' After he recovers from the surgery he's just, you know, he's our normal little boy, doing his stuff."

Most parents expressed satisfaction with the surgery and the functioning of their child's genitalia at present; however, they also noted concerns about the future: fearing negative physical, social, or emotional changes associated with puberty and adolescence, one parent stated "I'm more concerned with it as he gets older... what things are gonna look like... things are functioning perfect right now." Another parent noted "The physically hard part is... is done with, the surgeries are all done. I think now is the emotional." For most parents who continued to express concerns about their child's genital appearance or function following surgery, the worries were future-oriented.

**Figure 1:**
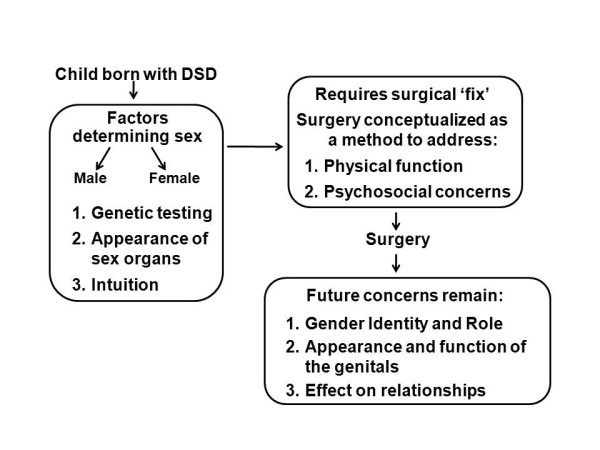
**Parents' perceptions of and experiences with the DSD decision-making process (gender assignment and genital surgery)**.

Parental concerns that persisted after surgery included (1) gender identity: "I worry that at some point he's going to feel like he's a woman trapped in a man's body, even though his female structures were removed," (2) gender role: "She throws in the 'Mom, will you paint my nails?' and I go fall over and do it right then and there... I dropped all that I was doing and painted her fingernails because she wanted to act like a little girl," (3) peers: "I worry he's going to be in gym class and people are going to notice things... I just always worry that, you know, that that will be frustrating for him to deal with in the future," (4) romantic partners: "The surgery can only fix so much, it's not going to look exactly normal. In the future, a husband or boyfriend may not be ok with it," (5) fertility: "[I worry about] him being able to have children and be able to feel that he is adequate," and (6) sexual orientation: "He's had so many problems. Is there a possibility of, you know, homosexuality?" No parent specifically stated that they regretted consenting to their child's surgery; however, one parent questioned the necessity in hindsight: "Did she even need to have that [surgery] in the first place? Should we have just left it alone?... It seems like with doctors, it's such a, like they just want to fix it and diagnose." Figure 1 schematically summarizes parent reports of experiences during the period of diagnostic evaluation and decision-making regarding genital surgery.

Despite parents' uncertainties and apprehensions about the future, parents in our study expressed confidence in the appropriateness of their child's gender assignment-including those whose children experienced either delayed gender assignment or reassignment. However, parents tended to express greater concerns about their child's gender development in cases in which the internal genital anatomy or sex chromosomes were discordant with the child's gender of rearing. Conversely, parents of children in whom these markers of sex development were concordant, despite an atypical genital phenotype (e.g., proximal hypospadias with bilateral undescended testes in child with 46, XY karyotype), expressed less concern over the child's gender development.

### Sharing Information

Although parents' concerns over their child's gender development depended in part on the specific DSD, this distinction did not predict the amount of information parents shared with others about their child's condition. Instead, comfort with disclosure depended on parents' outlook regarding: (1) the likelihood of stigmatization, (2) who they believed had the right to disclose information, (3) personal comfort in talking about anatomical aspects of DSD, and (4) parents' perceived ability to accurately explain DSD to others and/or have their child's condition understood.

*Concerns about talking with others*. Many parents expressed the view that sharing information about their child's condition would lead to stigmatization: "I don't want people to treat her different." Parents were also concerned about rumors and gossip: "I don't want rumors to start and for it to affect him later on in life-like socially because people don't understand the condition." Most fears were not based on direct experience; however, one parent reported a negative incident with a family member: "Her father came over and said that it wasn't his child because he don't make funny babies."

Several parents noted that, because DSD was potentially stigmatizing, they wanted to preserve their child's right to make decisions about disclosure: "There's a whole stigma associated with this, and it's unfortunate, and I have kept it mostly private for [my son] because I don't know how he wants to handle it when he gets bigger." Other parents, particularly those who opted for sharing more information, felt less need to defer the decision to their child: "I'm not embarrassed about what they have. You know, it's part of life."

Several parents felt their child's condition was extremely difficult to explain, and/or not something most people would understand. Additionally, parents were concerned that their attempts to explain the condition to others would generate more questions than they wanted to answer; one parent noted: "It is kind of exhausting trying to explain." Many parents expressed discomfort in talking to others about the anatomical aspects of their child's DSD: "It's a little bit of a personal area of the body so it's... I don't know. I don't want to run around with a banner saying that my child has an issue with that part of his body."

*Consequences of minimal sharing*. Most parents chose to limit the amount of information shared, with whom it was shared, and who was allowed to view their child's genitals. The practice of limiting information sharing proved to be difficult and stressful for many. In some cases, parents did not share details of the DSD with anyone, including close family (e.g., child's grandparents). Nevertheless, parents reported feeling pressured to talk about their child's condition with others: "People want to know, 'What's going on?' How he is. 'Why were you at the doctor?' So you kind of have to come up with a way to talk about it," or "I had to tell somebody, 'cause it was bursting inside of me. I'm like, I've gotta get somebody else's input or something." At the same time, parents believed that keeping their children's DSD private was extremely important: "It's hard having a child with something that you can't talk to people about. That you feel like you've got to have this huge secret all the time. That in itself is stressful in that you just can't tell people how you feel... [but] I can't jeopardize it, I can't take that risk. I just can't." Parents also reported avoiding situations in which their child's genitals might be seen: "I would change [my unaffected son] into a bathing suit at a side of a pool and not think twice about it and with [my affected son], I would *never *do that" or "I was like 'Oh no, you are not changing his diaper. I don't want to talk about it.'"

**Figure 2:**
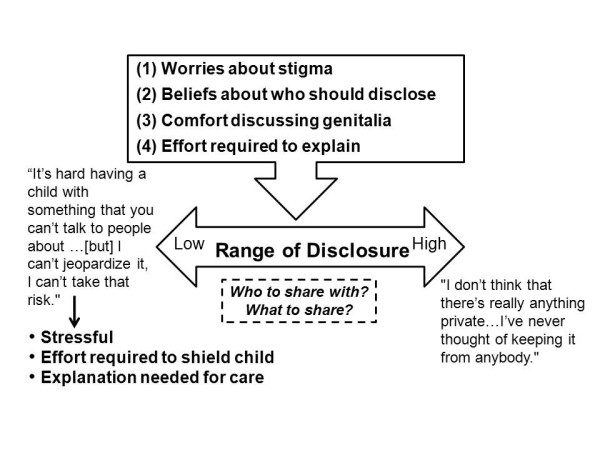
**Parents' decisions and experiences with sharing DSD-related information**.

Many parents decided to give others partial information about their child's DSD and its management in order to strike a balance between their desire to consult and share with others and their fear of negative consequences. One parent noted: "I guess it was a little bit easier to say, 'He had a bladder infection so we went to the doctor,' versus saying, 'Well, when he was born he had this birth defect and...'" More rarely, parents felt comfortable discussing the condition openly: "I don't think that there's really anything private... I've never thought of keeping it from anybody." Many parents reported that as time went on they became more comfortable with their child's condition and that sharing information was less pressing, particularly when their child was more independent (i.e., no longer in diapers and surgical aftercare was complete) or after genital appearance was modified. Figure 2 summarizes parents' experiences with disclosure of their child's DSD.

### Interactions with Healthcare Providers

*DSD education*. Many parents reported that until they received their child's diagnosis, they were unaware such conditions existed: "I didn't understand it. I'd never heard of it before." Parents felt that their unfamiliarity with their child's diagnosis contributed to their stress and feelings of isolation: "[I] had never talked to anybody that had ever experienced it and I kinda felt like... I was the only one ever having to deal with this." Parents often described gaining information from the medical team as helpful in decreasing this source of stress. Several parents mentioned physicians' drawings as a particularly helpful tool that enhanced their understanding.

Despite what parents believed were the providers' best efforts, some expressed frustration with the type or amount of information available to them: "The most stressful thing is just not being presented with clear-cut information." This same parent went on to explain: "[The medical staff] did give us pamphlets and they kind of explained things over and over, but you really want comprehensive information and it's hard." Still other parents noted positive experiences in obtaining information from their child's medical team, but encountered difficulties when seeking information through other sources: "The doctors don't overload you. They give you as much as you can handle, and go from there. The only problem is when you go home and you try and research this, and you get scared."

*Negative experiences*. Although the majority of parents described their communications with healthcare providers as supportive, a handful reported incidents described as "frustrating" or "exploitive." One parent said: "I felt like [the doctor] was looking just to look... I felt like he was just exploiting her for his own... you know--he wanted to see the surgery." While parents expressed an understanding that a hospital may also have a teaching mission, they did not feel that this justified the large number of providers interacting with them or their child or the repeated examinations of their child's genitalia: "There were so many residents and different people coming in at every time it felt like he was a show-horse and that was frustrating... you're trying to breastfeed for the first time."

## Discussion

For families, the birth of a child with a DSD and the attendant uncertainty about the child's gender and future psychological and psychosexual development is believed to be extraordinarily stressful [28]. High levels of stress are likely to arise from both the unfamiliarity and perceived stigma associated with DSD and from the controversies surrounding current clinical management of these conditions. Despite parents having had access to services delivered at reputable tertiary care facilities, parents' experiences suggest a need to continue to strengthen the Consensus Statements' call for comprehensive and integrated long-term care for families affected by DSD [1]. The findings suggest, in particular, a need to improve provider-family/patient communication and to increase the availability of psychosocial support as an integral component in the delivery of care. While the parents in this study reported that early surgical interventions eased some of their immediate concerns, the findings suggest varied opportunities for enhancing education, shared decision-making, and linking families of affected children to others sharing common challenges [1].

Study participants expressed certainty about their child's sex and gender-whether announced at birth or subsequently assigned. For those parents that referenced diagnostic tests as a source of their certainty, it is unclear if such confidence stemmed from their understanding of test findings (e.g., karyotype, studies of internal reproductive anatomy), or if these results were selectively called upon to reinforce their intuitions. Several parents' reports suggested that healthcare providers framed clinical and laboratory findings to either generate or reinforce parents' beliefs that these indicators of sex development are determinative of gender identity. Until the mid-1950s, medical management of persons affected with DSD was guided by the belief that an individual's ''true sex'' could be revealed through examination of internal anatomy and that the person's identification as boy or girl would naturally conform to their ''true sex" [28]. We now know that individual markers of biological sex can be associated with a range of gender outcomes [29-33]. Given the potential effect of this information on parental decision-making, this prompts the question to what degree and with how much detail should healthcare providers, in the promotion of the principles of shared decision-making, educate parents about the nuances of somatic sex development and their inconsistent relationship with gender identity in DSD if, by doing so, they potentially enhance cognitive and emotional conflict in the parents? Understandably, providers may fear alienating those parents who already have a strong conviction about their child's sex and gender by presenting a contrary viewpoint. However, without providing comprehensive information, providers risk breaching the ethics of informed consent for clinical interventions and the possibility that the parents will later learn about the withheld information and interpret provider's selectivity as a shortcoming or even a deception.

Delivering information that aligns with the ethics of informed consent is particularly critical when interventions are elective, non-urgent, controversial, and associated with potentially serious risks [34,35]. To enhance transparency and diminish the likelihood of decisional regret, Karkazis and colleagues recently outlined a 6-step model for shared decision-making in DSD as it pertains to genital surgery in young children [34].

Parents in our study also reported frustration over gaps in information about their child's condition. This could be due to a number of factors: (1) uncertainty about the diagnosis in the early stages which creates difficulties when discussing the condition, its course, and early management, (2) lack of educational tools that make complex medical concepts accessible to the general public, (3) parents' potentially diminished capacity to process complex medical concepts and make decisions during a time of stress, and (4) the existing gaps in medical literature surrounding DSD and DSD care. Parenting with uncertainties regarding the child's future is common in pediatric chronic illness and disease specific parent-to-parent support has been shown to be particularly useful in helping parents to cope with uncertainties and their frustrations during early decision-making; the use of support groups is additionally endorsed by the Consensus Statement [1]. Development of high quality DSD-specific educational content that adheres to the principles of health literacy [36] may also facilitate improved communication and knowledge sharing between provider and family. One exemplary sample of such content are the *Sex Development *pages of the *AboutKidsHealth *website edited by faculty and staff at the Hospital for Sick Children in Toronto [37]. Providers may also integrate HRQoL assessments into clinical care as a means of better identifying and addressing parent and child needs and concerns [29].

Parents recalled events surrounding their child's genital surgery with particular salience. Consistent with other reports, parents viewed genital surgery as a necessary and obvious "fix" for their child's DSD [9,13,38]. They justified early surgery as a means of averting negative consequences, such as stigmatization, that are associated with atypical genital appearance or function [13,39,40]. While satisfied with surgical outcomes, they continued to be concerned about the child's future experiences. Parents' worries primarily pertained to uncertainty about changes in their child's genital appearance or function associated with the onset of puberty, whether spontaneous or by hormone replacement, and the renewed risk of stigmatization or rejection by potential sexual/romantic partners and peers. These findings point out that early surgery reduces early parental concerns regarding genital appearance, but does not eliminate worries about their child's future sex development or function. Accordingly, there is an important need to maintain contact with families in order to monitor parents' expectations and address unresolved anxiety about the child's future, even in those cases in which early surgery was considered an unqualified success.

Previous research regarding disclosure in DSD has focused on harm to affected individuals by being either uninformed or misinformed about their condition [1,6,15]. However, parents of affected individuals also appear to grapple with issues of information sharing. For the parents in the present study, withholding information from other adults was motivated by a desire to protect the child's privacy and to prevent stigmatization. However, parents varied markedly in the degree to which they disclosed details. Those who maintained fairly strict privacy experienced this approach as very stressful, whereas those who chose to share information with trusted others reported experiencing less strain. The current findings suggest that in addition to parents educating their child in a developmentally-appropriate way about their condition, they may benefit from more explicit and extensive discussions about sharing information with their usual social support system (family and friends). The extent to which such discussions between parents and providers are currently occurring is unclear. The results suggest, however, that the status quo is inadequate with respect to the counseling of parents on the challenges of information sharing and support seeking. The child's right to privacy should be balanced against the risks associated with secrecy, promoting a sense of shame, and limiting opportunities for social support. Failure to achieve this balance could contribute to unresolved parental feelings of guilt and possibly to a negative self-concept, shame or isolation for the DSD-affected person [9,41,42]. The Consensus Statement [1] identifies the timing and content of information management as warranting targeted study.

The majority of interactions between parents and their child's healthcare providers were described as positive. However, several negative interactions were noted in the context of genital examinations which parents felt were unnecessary or exploitative. There is reason to be concerned that repeated genital examinations and medical photography can have lasting and severe negative psychological consequences [43-45]. Responses to genital examinations in DSD and strategies to perform them in a way that reduces the likelihood of harm is another area in which systematic information is missing. In the interim, providers should continue to communicate openly with the patient and family, describe the purpose of the exams, ask for consent (and when appropriate, assent), and minimize patient exposure. Input from child life specialists who are trained to mitigate distress associated with medical procedures may be helpful [46].

### Study Strengths and Limitations

This study presents the experiences of a relatively large sample of mothers and fathers of diagnostically diverse young children with DSD. Parent participants were identified through a systematic review of the children's medical records; only a small proportion of those contacted refused participation. An additional strength of this study was the extent of fathers' participation (35%). Frequently, studies of children with medical conditions rely exclusively on maternal reports [47,48].

All children had undergone genital surgery. It is possible that this high rate of genital surgery is related to our sampling process which identified participants via chart review at academic medical centers. This sampling approach may be suboptimal for ascertaining cases in which surgery had not been performed. The proportion of children with DSD who have not undergone surgical interventions is unknown. Accordingly, it is difficult to determine the extent to which our findings can generalize to patients and families who have elected not to have surgery performed. Research by Warne and Raza [49] encourage investigators in this area to be sensitive to variability across cultural and socioeconomic contexts.

Interviews were guided by the standardized probes to evaluate the quality of items to be incorporated into HRQoL questionnaires for parents of young children with DSD. Accordingly, although parents were asked to talk about areas of importance to them and their family that were not specifically covered in the HRQoL instrument, there may be topics of importance that did not emerge due to the interview structure. Tempering this concern is that novel questions were added to the HRQoL questionnaires based on ideas parents generated during the interviews, demonstrating that parents explored experiences beyond the confines of the cognitive interview structure. Finally, because interviews were conducted at varying intervals after some of the events being described, the potential exists for distorted recall.

## Conclusions

Parents expressed a strong desire for their children's lives to be as "normal" as possible. In order to do what was best for their child with DSD, parents sought definitive information and guidance on management. Our findings suggest that parents did not always have all the information they wanted, when they wanted it, or in some cases, an accurate understanding of available information or sufficient awareness of the gaps in research on outcomes. Occasionally, parents' communication with providers was remembered and described as intentionally encouraging an oversimplified picture of DSD and factors influencing outcome or, alternatively, that parents selectively incorporated information. Parents rationalized genital surgery as a "fix" for atypical appearance, function, and psychosocial concerns, despite a lack of empirical evidence indicating that surgery can fully address all of these challenges. Although parents reported being less concerned with the immediate implication of their child's DSD post-surgery, they remained concerned about their child's future adaptations.

The strains that parents experienced were, in some cases, ameliorated by the support of trusted family and friends. However, not all parents availed themselves of this coping strategy, viewing disclosure as too risky. This latter subgroup may be in greatest need of support from behavioral health members of the DSD interdisciplinary team [50-53].

Overall the findings suggest that families affected by DSD may benefit from enhanced adherence to the guidelines of shared decision-making, increased efforts to provide information objectively in line with the ethics of informed consent, and early and ongoing inclusion of behavioral healthcare providers in interdisciplinary teams caring for affected families.

## List of Abbreviations

DSD: Disorders of Sex Development; HRQoL: Health Related Quality of Life.

## Competing interests

The authors declare that they have no competing interests.

## Authors' contributions

HPC contributed significantly to the qualitative methods/secondary analysis research design; MG and DES supervised the design. HPC, LW, and MC executed the qualitative content analysis; MG and DES supervised the analyses. MG conducted the majority of interviews; AS and DES conducted a substantial proportion; ALQ conducted one in addition to training interviewers. LW conducted the five member-checking interviews and their analysis. HPC, LW, MG, and MC transcribed interviews. MG collected all questionnaire, demographic, and medical chart excerpt data; maintained the databases; conducted descriptive statistical analyses. MG, ALQ, BK, and DES conceived of the parent study and participated in guiding study design and execution. AS and BK excerpted medical charts. HPC, LW, MG, and DES contributed significantly to manuscript drafts; MC contributed significantly to early drafts. All authors contributed comments throughout the writing; all read and approved the final manuscript.
